# Assessment of drowning status based on age groups in the population covered by Isfahan ‎University of Medical Sciences from 2012 to 2017‎ 

**Published:** 2019-08

**Authors:** Mahshid Ahmadian, Amin Esmaeili, Leyla Rasti

**Affiliations:** ^*a*^Director of the Department of Non-Communicable Diseases of Isfahan Province, Iran.; ^*b*^Disaster and Accident Specialist, Isfahan Province Health Center, Iran.; ^*c*^Specialist in Genetics of Isfahan Health Center, Iran.

## Abstract

**Background:**

According to reports from the World Health Organization, drowning is the third cause of death ‎due to unintentional accidents and accidents, which is high among children under the age of 5 ‎years. In addition, a large number of children who survive the risk of drowning endure long-‎term outcomes and disabilities, which impose a significant spiritual and material burden on ‎families and the health system of countries. Over the past year, according to a report from the ‎Forensic Medicine Organization, 1,269 people died of drowning, of which 858 were male and ‎‎168 were women. In this regard, information collected from the one-to-59-month-old children's ‎care system in 1396 shows that 12 percent of deaths from unintentional accidents in this age ‎group are drowning. The review of texts and articles suggests that the implementation of ‎existing laws or regulations, or the formulation of new regulations on the optimization of the ‎environment in pools, wells and fish farming ponds, and enhancing safety within the seaside, ‎lakes and rivers' seabeds, on education of the people , Will be very effective. ‎

**Methods:**

A cross-sectional study was carried out using data recording software and death records. Data ‎was analyzed using SPSS software.

**Results:**

Based on the information available in the accident registration software, during the years 2012 ‎to 2017, 606799 people were referred to hospitals affiliated to Isfahan University of Medical ‎Sciences due to accidents, of which 190 were drowned, of which 63 (33.2%) have died. Also, ‎according to this information, the most common occurrence is drowning in the age group of 18 ‎to 29 years, which is 29.47% of all deaths due to drowning in 2012-2017. The second rate of ‎death from drowning is 27.3% for the age group of 30 to 59 years, and the next rank with the ‎percentage of 26.31 for the age group of 6 to 18 years and 13.15% for the age group under 5 ‎years.‎

**Conclusions:**

The findings of this study suggest that the following measures can be taken to prevent and ‎reduce drowning events: Holding the Supreme Committee of the Safe Community of the City ‎and the above plans, a joint meeting with program-related organizations 3. Conduct training ‎sessions to prevent drowning in the public, Supervise related sites (swimming pools, etc.) ‎regarding the supply of equipment and emergency shelter, Co-ordination with municipalities for ‎the repair and safety of ponds and ponds in parks and other public places, First aid training ‎through holding training and distributing pamphlets and tracks, Notification via audio and ‎video, Co-ordination with agriculture Jihad for the safety of canals and agricultural land ponds, ‎Co-ordination with the Water and Wastewater Authority and the municipalities to secure the ‎wells and Installing signs and warning signs along rivers and canals and Immunization of baths ‎Pools and ponds in the home.‎

**Keywords:**

Drowning-age groups, Software, Recording of accidents, Death record software

